# The Orai-1 and STIM-1 Complex Controls Human Dendritic Cell Maturation

**DOI:** 10.1371/journal.pone.0061595

**Published:** 2013-05-20

**Authors:** Romain Félix, David Crottès, Anthony Delalande, Jérémy Fauconnier, Yvon Lebranchu, Jean-Yves Le Guennec, Florence Velge-Roussel

**Affiliations:** 1 EA 4245 Cellules Dendritiques, Immunomodulation et Greffes, Université François Rabelais, IFR-136 Agents Transmissibles et Infectiologie, UFR de Médecine, Tours, France; 2 Centre de Biophysique Moléculaire CNRS UPR 4301, Orléans, France; 3 Institut National de la Santé et de la Recherche Médical U930 Imagerie et Cerveau, Equipe 5, Tours, France; 4 Institut National de la Santé et de la Recherche Médical U637, Physiopathologie Cardiovasculaire, Montpellier, France; 5 Service de Néphrologie et d'Immunologie Clinique, CHRU Tours, Tours, France; 6 UFR des Sciences Pharmaceutiques, Tours, France; National Cancer Institute (INCA), Brazil

## Abstract

Ca^2+^ signaling plays an important role in the function of dendritic cells (DC), the professional antigen presenting cells. Here, we described the role of Calcium released activated (CRAC) channels in the maturation and cytokine secretion of human DC. Recent works identified STIM1 and Orai1 in human T lymphocytes as essential for CRAC channel activation. We investigated Ca^2+^ signaling in human DC maturation by imaging intracellular calcium signaling and pharmalogical inhibitors. The DC response to inflammatory mediators or PAMPs (Pathogen-associated molecular patterns) is due to a depletion of intracellular Ca^2+^ stores that results in a store-operated Ca^2+^ entry (SOCE). This Ca^2+^ influx was inhibited by 2-APB and exhibited a Ca^2+^permeability similar to the CRAC (Calcium-Released Activated Calcium), found in T lymphocytes. Depending on the PAMPs used, SOCE profiles and amplitudes appeared different, suggesting the involvement of different CRAC channels. Using siRNAi, we identified the STIM1 and Orai1 protein complex as one of the main pathways for Ca^2+^ entry for LPS- and TNF-α-induced maturation in DC. Cytokine secretions also seemed to be SOCE-dependent with profile differences depending on the maturating agents since IL-12 and IL10 secretions appeared highly sensitive to 2-APB whereas IFN-γ was less affected. Altogether, these results clearly demonstrate that human DC maturation and cytokine secretions depend on SOCE signaling involving STIM1 and Orai1 proteins.

## Introduction

Dendritic cells are highly specialized antigen presenting cells (APC) able to induce both specific immunity and immune tolerance. Because of these properties, they represent the most important regulating cells of the immune system [1], [2]. On the basis of information gathered from the tissue where they reside, DC adjust their functional activity to ensure that protective immunity is favored while unwanted or exaggerated immune responses are prevented. Immature DC become mature DC with functional competences in response to a number of stimuli through numerous cell surface receptors such as cytokine receptors, TLR (Toll-like Receptors) or type C lectins [3], [4]. It results in a sustained increase of intracellular Ca^2+^ acting as a second mediator for T and B cell receptor signaling leading to gene activation, cellular proliferation and cytokine secretion [5], [6]. The process of DC maturation induced by extracellular ligands plays a key role in the immune responses but the calcium signaling mechanisms involved in this phenomenon are not well understood. A previous study suggested that a Ca^2+^ influx (also named CCE, SOCE or CRAC) might be involved in murine DC maturation [7]. Among different modalities of Ca^2+^ entry, the implication of an ion channel (selective to Ca^2+^) sensitive to endoplasmic reticulum (ER) Ca^2+^ stocks, named SOC was shown to control this influx. SOCs can be activated by ER Ca^2+^ depletion leading to a Ca^2+^ influx response named SOCE (Store-Operated Calcium Entry). This SOCE allows reloading of ER Ca^2+^ stocks and is involved in the regulation of several physiological functions [8].

Although their presence and their functionality were investigated in murine DC, there are very few data regarding the role of Ca^2+^ homeostasis and the role of ion channels in human DC. The use of a calcium ionophore induced a mature phenotype in human DC [9], as well as an inhibition of IL-12 secretion [10]. Bagley et al. showed the involvement of PLC (Phospholipase C) in the maturation process of DC induced by LPS [11]. Indeed, PLC activation induced Inositol Triphosphate (IP_3_) synthesis, which in turn induced ER Ca^2+^ stocks release through activation of IP_3_ Receptor (IP_3_R).

Store-operated Ca^2+^ entry (SOCE) appears to be the main mechanism used by many cell types to initiate signal transduction [12]. We choose to focus on two recently discovered molecules: Orai1 and STIM-1 [13]. These two proteins, as they move close to MHC complex in the immunological synapse, are up-regulated during T cell activation and provoke cell polarization [14], [15]. One of the implications of Orai1's and STIM-1's up-regulation in the early stages of the immune response is to amplify and ensure CRAC channel-mediated Ca^2+^ signaling for clonal expansion, differentiation and calcium-dependent regulation of gene expression in T cells. Severe combined immunodeficiency (SCID) patients have been associated with lack of both functional ORAI and STIM-1 [16], [17]. Although their presence and their functionality were investigated in mouse DC, there are very few data regarding the role of these proteins in Ca^2+^ homeostasis and the role of ion channels in human DC.

These data clearly indicate that Ca^2+^ signaling via the Orai1/STIM1 complex is likely to be of importance in immunological regulation. We aim to investigate the mechanisms by which Ca^2+^ participates in DC maturation. To do so we analyzed early events of DC maturation process and functional consequences of its inhibition.

## Materials and Methods

### 1. Generation of Dendritic Cells from Peripheral Blood Mononuclear Cells (PBMC)

Healthy volunteer's blood was obtained by cytapheresis after signed informed consent managed by the French Blood Department. PBMCs were isolated by Ficoll Hypaque density gradient centrifugation (density 1.077, Lymphoprep; Abcys SA, France) and resuspended in culture medium (RPMI, 10% SVF, 1% Penycillin-Streptomycin, 1% L-Glutamine) after 2 washings. Monocyte-derived dendritic cells were prepared after selective adhesion of monocytes to plastic as previously described [18]. Briefly, PBMC were incubated for 45 min at 37°C in 5% CO2 in culture flasks (100 million in a 75 cm^2^-Falcon flask, Becton Dickinson, Mountain View, CA, USA), non-adherent cells were discarded and medium containing rhGM-CSF (1000 U/ml) and rhIL-4 (25 ng/ml) was added. Cells were suspended in culture medium (containing 1000 U/ml rhGM-CSF and 25 ng/ml rhIL-4). On day 5, immature DC were harvested (DC-SIGN+/HLA-DR+ >95%), washed and suspended in culture medium with IL-4 and GM-CSF.

### 2. Cell treatment

DC were cultured during 18 hours in the presence of several concentrations of Ca^2+^ as following; RPMI at 0.4 mM, RPMI with BAPTA 10 mM to obtain Ca^2+^ free solution, RPMI with CaCl_2_ 2 mM and RPMI with CaCl_2_ 4 mM. TLR agonists (LPS (50 ng/ml), zymosan (25 µg/ml) and TNF-α (20 ng/ml)) as well as Ca^2+^ inhibitors such as Thapsigargin (750 nM), D609 (100 μM) and 2-APB (100 μM) were added directly to immature DC for 18 hours in individual wells. All drugs and chemicals were purchased from Sigma-Aldrich (St Quentin Fallavier, France). Cells were harvested, washed and used for analyses in flow cytometry, mixed culture reaction or spectrofluorometric analysis. For reagents suspended in dimethyl sulfoxide (DMSO), an equal volume of DMSO was added to control cultures. At the concentrations used, DMSO has no inhibitory or stimulatory effects on DC (data not shown).

### 3. Solutions

The physiological saline solution (PSS) had the following composition (in mM): NaCl 140, KCL 5.4, MgCl_2_ 1, NaH_2_PO_4_ 0.33, CaCl_2_ 1.8, D-Glucose 11.1 and HEPES 10; adjusted to 7.4 with 1 M NaOH. The Ca-free solution had the following composition (in mM): NaCl 140, KCL 5.4, MgCl_2_ 1, NaH_2_PO_4_ 0.33, EGTA 1, D-Glucose 11.1 and HEPES 10; adjusted to 7.4 with 1 M NaOH. Thapsigargin (TG), 2-ABP (2-AminoPhenyl Borate), LPS (Lipopolysaccharide), zymosan and TNF-α were added to the PSS at concentrations indicated in the figure legends.

### 4. Fluorescence measurements

Intracellular Ca^2+^ concentrations were estimated using the ratiometric fluorescent dye Fura-2. Immature DC were plated on cover slips (Fluorodish FD35-100, WPI, UK) coated with poly-L-Lysin (Sigma-Aldrich, St Quentin Fallavier, France) in PSS. Cells were incubated in PSS containing Fura-2 AM (5 µM) (Sigma-Aldrich, St Quentin Fallavier, France), the membrane-permeant acetoxymethyl ester form of Fura-2, diluted in pluronic acid-F127 (Sigma-Aldrich, St Quentin Fallavier, France), during 60–75 min at 37°C. Cells were then washed with PSS and left for 2 additional minutes before recording.

Samples were analyzed with a Nikon Eclipse TE2000-S inverted epi-illumination microscope (Nikon, France). The excitation light source was a 75-W Xenon arc lamp. Excitation light at the two-excitation wavelengths maxima of Fura-2 (340/380 nm) was chopped by a monochromator (Cairn Optoscan, UK). The excitation protocol was a 50 ms excitation at each wavelength every 2 s. Excitation light was directed through a 60× oil immersion objective with a numerical aperture of 1.4 (Nikon Plan Apo, France). Fluorescence emissions at 510±20 nm were detected by a photomultiplier tube (PMT) placed on the side of the microscope. The analogical signals of PMT were digitized by a Digidata 1322, a converter (Axon Instrument, USA) at a sampling frequency of 2 kHz. This numeric signal was analyzed using Clampex 8.2 (Axon Instrument, USA). Background fluorescence was determined at 340 and 380 nm from an area of the dish free of cells after experiment and was routinely subtracted.

### 5. Flow Cytometric Analysis

Dendritic cells were incubated at 4°C with saturating concentrations of fluorochrome-conjugated mAbs (CD80, CD86, CD83, CD25, HLA-DR and DC-SIGN) in the dark for 30 min, washed twice with PBS, fixed in 0.5% PFA-PBS solution and analyzed with a FACSCanto (Becton Dickinson, France). Dead cells were gated out on the basis of their light scatter properties. Data were analyzed with Diva Software.

For intracellular detection of IFN-γ, cells were incubated in Golgistop solution (Becton-Dickinson) for the last 5 hours of culture; next the cells were permeabilized with BD cytofix/cytoperm solution (Becton-Dickinson, France) and finally labeled with an APC-coupled antibody specific for IFN-γ (Becton-Dickinson, France) according to the manufacturer's instructions.

### 6. Enzyme-linked immunosorbent assays (ELISA)

Cell culture supernatants were harvested and stored at −80°C until assayed for cytokines. Human IL-10, IFN-γ and IL-12 p70 concentrations were measured by ELISA using Ready-Set-Go kits from e-Bioscience (Montrouge, France) according to the manufacturer's instructions. Briefly, microtiter plates were coated overnight at 4°C with antibodies specific for IL-12p70, IL-10 and IFN-γ, from e-Bioscience (Montrouge, France). The plates were washed and blocked according to the manufacturer's instructions. Samples and standards were analyzed in triplicate, and tested with the avidin-peroxydase system. Optical densities were measured in an ELISA plate-reader at 450 nm wavelengths.

### 7. RNA extraction and Real-time PCR

Total RNA was isolated using RNA extraction minikit (Qiagen, Germany) following the manufacturer's protocol. RNA yield and purity were determined by spectrophotometry, and only samples with an A260/A280 ratio above 1.6 were kept for further experiments. Prior to reverse transcription, total RNA was treated with DNAase I for 30 min at room temperature. RNA was then reverse-transcribed using Superscript II reverse transcriptase and oligodT (Invitrogen, France). Quantitative (real time) PCR experiments were performed with a Lightcycler 480 (Roche, France). The PCR protocol consisted in a denaturizing step at 95°C for 2 min, followed by 35 cycles of amplification at 95°C for 15 s, 60°C for 30 s, and 72°C for 10 s. The experiments were performed in duplicate, and negative controls containing water instead of first strand cDNA were done. The results were calculated with the ΔCt method, where the parameter Ct (threshold cycle) is defined as the fractional cycle number at which the PCR reporter signal passes a fixed threshold. Primers used for PCR experiments had the following sequences (expected sizes): STIM-1, forward 5′-GCATCTTGCCTGGAGACCGT-3′ and reverse 5′-CAAGACGGACGCATACATCC-3′; ORAI1, forward 5′- GTCACCTACCCGGACTGGAT-3′ and reverse 5′- TGGAGGCTTTAAGCTTGGCG-3′. In order to prevent amplification of genomic DNA, forward and reverse primers used spanned neighboring exons.

### 8. Immunofluorescence and confocal analysis

For immunofluorescence labeling of STIM1 and Orai1, DC were spun on glass LAB-TEK (Nunc International, USA) coated with Poly-L Lysine (Sigma-Aldrich, France). Cells were permeabilized and fixed by BD Cytofix/Cytoperm ^TM^ Plus kit (BD Biosciences, USA) in labeling medium (PBS with 4% FBS and 0,1% Azide) at room temperature. After fixation, samples were then incubated with purified antibodies diluted in labeling medium during 1 hour at room temperature. As primary reagent, we used as primary antibody a monoclonal mouse anti-human STIM-1 (Santa Cruz USA) and polyclonal rabbit anti-human Orai1 (Santa Cruz, USA). After washing cells twice with labeling medium, specific staining was detected by Alexa Fluor 488 conjugated goat anti-mouse IgG and Alexa Fluor 564 conjugated goat anti-rabbit IgG (Invitrogen, France), for STIM-1 and Orai1 respectively. Cover slips were placed in mounting medium (Invitrogen, France) and visualized with a LMS510 meta Zeiss confocal microscope equipped with a x63 water immersion objective (NA: 1. 2). Images shown are single optical slides, with the double labeling.

### 9. RNA interference

Pre-designed si-RNAs (Santa Cruz Biotech, USA) specific for STIM1 or Orai1 were used to inhibit STIM1 and Orai1 expression. In each experiment, 4.10^5^ immature DC were plated in 24-well plates in 400 µl growth medium (Opti-MEM containing 10% FBS without antibiotic). Lipofectamine RNAi-Max (1 µl/well; Invitrogen, France) was first diluted in 50 µl Opti-MEM (Invitrogen, France) for 5 minutes before being mixed with an equal volume of Opti-MEM containing 20pmol of siRNA. After 20 minutes, 100 µl of the Lipofectamine/siRNA mixture were added to the cells to perform siRNA transfection. Fresh growth medium (500 µl) was added 4 hours after transfection. Cells were cultured for 36 hours at 37°C to obtain optimal silencing of targeted genes. The efficiency of gene silencing was evaluated by western blot and flow cytometry. A siRNA Control (Santa Cruz Biotech, USA) was tested as negative control. The sequence of this siRNA Control is not specific to any genes. Interference efficiencies were evaluated by western blotting using a monoclonal antibody anti-STIM-1 (clone H180) and polyclonal anti-Orai-1 (clone H46) from Santa Cruz Biotechnology.

### 10. Statistical analysis

Statistical analyses were performed using a nonparametric Mann – Whitney test with XLSTAT 2007 software. For all tests, a *p-*value of <0.05 was considered statistically significant.

## Results

### 1. Extracellular calcium induces mature DC phenotype

**Figure 1:**
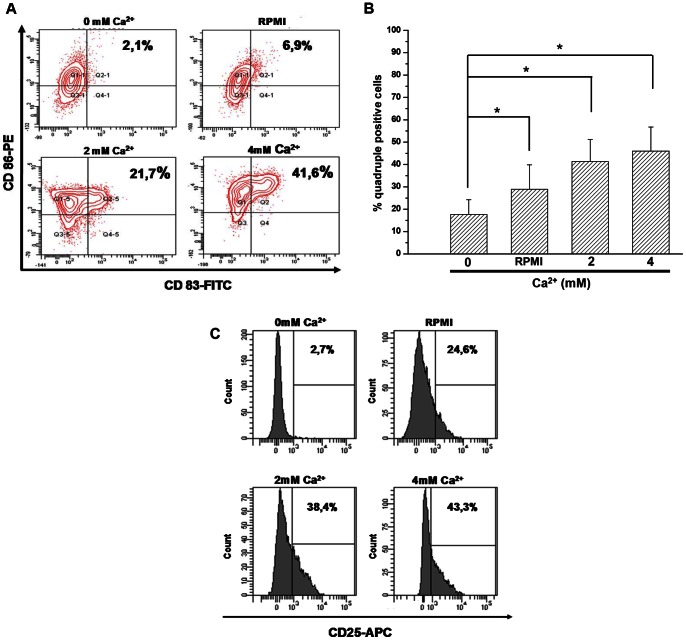
Expression of maturation markers in human dendritic cells according to extracellular Ca^2+^ concentration. DC were cultured with increasing concentrations of Ca^2+^ **(in mM)**. Cells were harvested and double or quadruple staining was assessed by FACS analysis for the 4 conditions. **A** – *CD83 expression is increased by extracellular Ca2+ concentration*. The population showed on this panel is gated on DC-Sign labeling. The percentage of double (CD86 and CD83) positive cells is shown in panel A. Results are representative of 7 independent experiments. **B** – *Expression of maturation markers in relation to extracellular Ca^2+^ concentrations*. The same population expressed CD80, CD86, HLA-DR and CD83 in increasing proportions in increasing extracellular Ca^2+^ concentration. The percentage of quadruple positive cells is shown in panel B. Graph bars represent the mean of 6 independent experiments (mean ± SD, *p<0,05). **C** – *CD25 expression in relation to extracellular Ca^2+^*. Grey histograms represent the expression of the cell-surface marker CD25. Results represent one out of 7 independent experiments.

We evaluated the expression of DC cell surface markers of maturation in presence of different calcium concentrations by flow cytometry. An increased extracellular Ca^2+^ concentration resulted in an increase of expression of CD83 and CD86 (Fig. 1A). We analyzed the percentage of DC quadruple positive cells (CD86/HLA-DR, CD80, CD83) in Fig. 1B. This percentage increased in parallel with the extracellular Ca^2+^ concentration (from 2 to 4 mM). The surface expression of CD25 increased from 24.6% to 43.3% when the extracellular Ca^2+^ concentration increased from 0.4 to 4 mM (Fig. 1C). In comparison, only 2.7% of DC expressed CD25 at their surface in the absence of Ca^2+^.

### 2. CRAC channel-mediated Ca^2+^ influx promotes DC maturation

**Figure 2:**
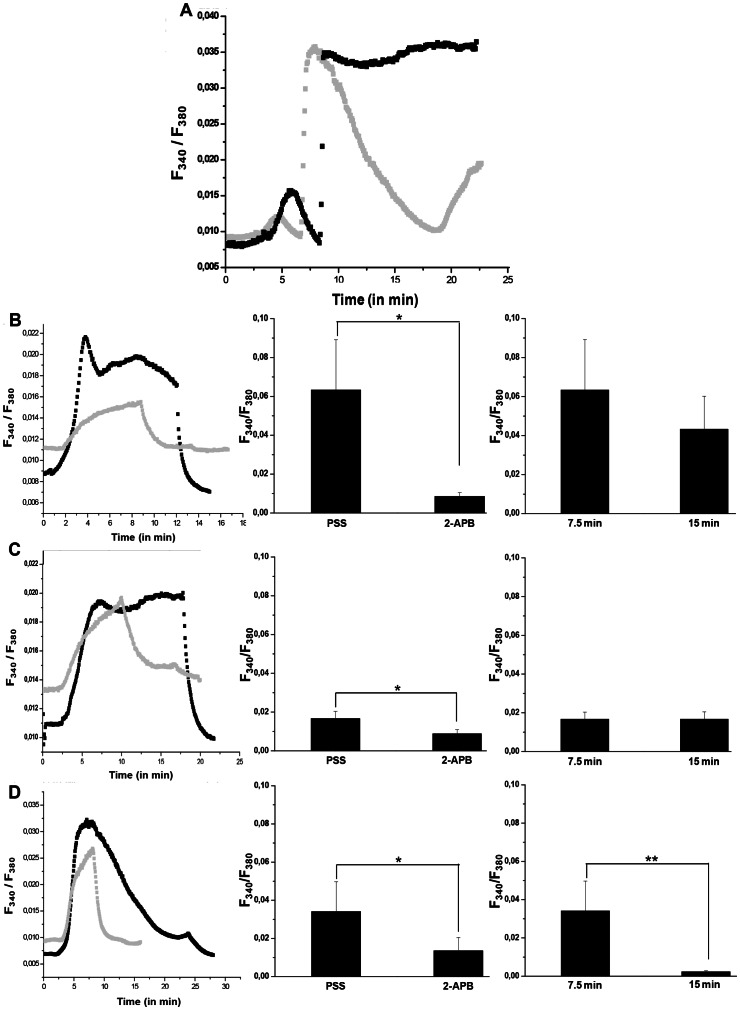
Identification of SOCE during the earlier events of DC maturation. DC were treated with thapsigargin (TG) in calcium free PSS, then PSS supplemented with 2 mM Ca^2+^ was added (**A**). 100 µM 2-APB PSS solution induced a rapid and reversible decrease of [Ca^2+^]_i_ (Grey trace). According to different maturation signals (LPS, **B**; TNF-α, **C** or zymosan, **D**), the variations of [Ca^2+]^_i_ in the presence of SOCE inhibitor (Grey trace, at 100 µM 2-APB) or its absence (Black trace) were observed by microspectrofluorimetry on left panels. Results are representative of 7 independent experiments. Central panels represent the maximal amplitude mean of CCE according to the treatment. Right panels represent the CCE amplitude mean at 7.5 and 15 min. Means were obtained from 7 independent experiments.

Since we demonstrated that extracellular calcium was able to modify the expression of phenotype markers in DC, we hypothesized that a calcium entry occurs during the earlier events of DC maturation. In Ca^2+^ free solution, TG induces store depletion. Calcium reintroduction induces a CCE sensitive to 2-APB. So, a SOCE following calcium store depletion (from ER stocks) was observed in human DC after application of TG, and it was sensitive to 2-APB (Fig. 2A). In addition, DC stimulation with TLR agonists resulted in a rapid and sustained increase in [Ca^2+^]_i_ suggesting that maturation was accompanied by CRAC channels activation (Fig. 2B, 2C and 2D). PSS solution perfusion with 100 µM 2-ABP significantly reduced but did not fully eliminate [Ca^2+^]i increase following LPS, zymosan or TNF-α treatments. For LPS, Ca^2+^ entry presented higher amplitude compared to those observed with zymosan and TNF-α (0.061 versus 0.034 and 0.018). Moreover, the continuous component in SOCE was sensitive to 2-APB after 10 min (Fig. 2B and 2C). In contrast, only a transient and reversible increase of [Ca^2+^] was observed in zymosan condition (Fig. 2D). A decrease of the zymosan-induced SOCE amplitude was observed in presence of 2-APB with a lesser effect compared to LPS and TNF-α (0.016 versus 0.008 and 0.009) (Fig. 2D versus 2B and 2C).

### 3. Both maturation and cytokine secretions depend on CRAC channels in DC

**Figure 3:**
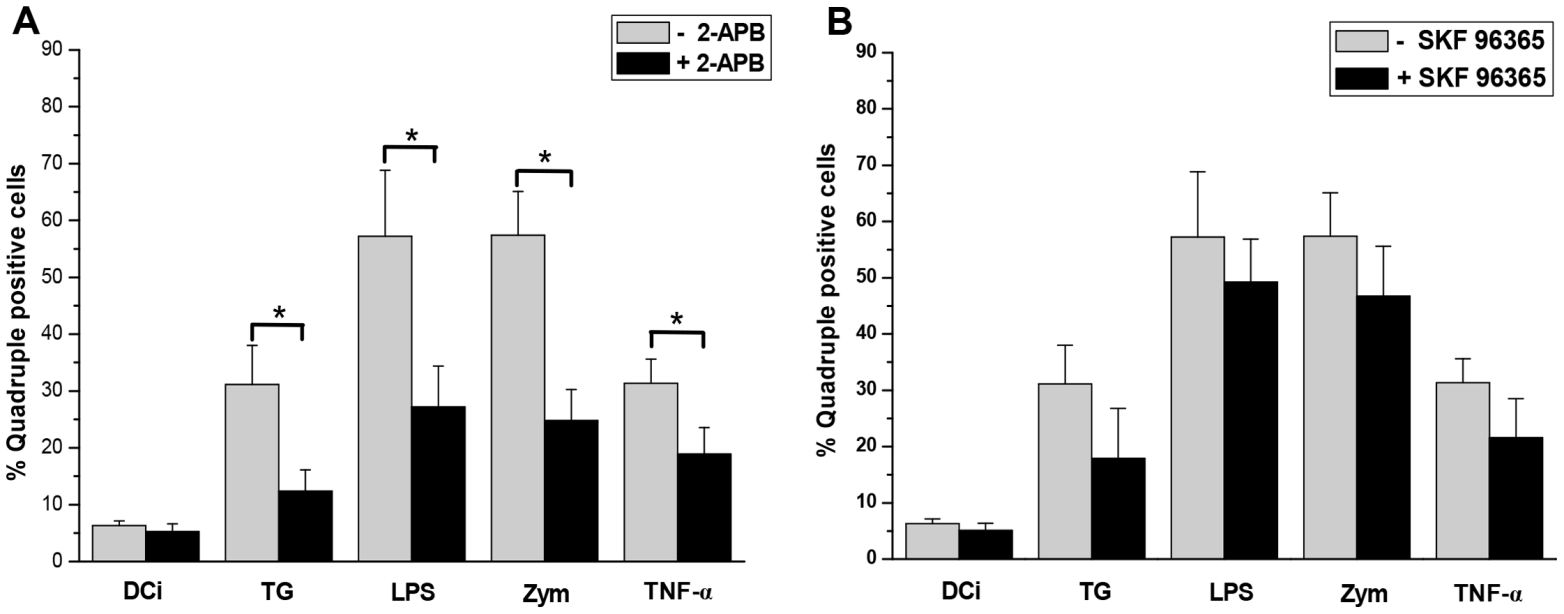
2-APB decreased the expression of maturation markers in human DC. In panel **A**, DC were cultured in medium with maturating agents (TG (750 nM), LPS (50 ng/ml), Zymosan (25 µg/ml) or TNF-α (20 ng/ml)) in the absence or presence of 2-APB (mean of percentage ± SD, *p<0,05, n = 7). In panel **B**, DC were cultured in medium with the maturating agents in the absence or presence of SKF 96365 (100 µM) (n = 7 for DCi, TG and LPS conditions and n = 4 for Zymosan and TNF-α). The marker expressions were analyzed by FACS as described for figure 1B. In panel C, ORAI-1 and STIM-1 protein expressions were analyzed by western blotting to control Si-RNA efficacy.

In DCi, quadruple positive cells represented 6.3% of the DC population. During the next 18 hours of culture in presence of different maturating agents, the expression of these markers increased up to 31.1% with TG, 57.2% with LPS, 57.3% with zymosan and 31.4% with TNF-α treatment (Fig. 3) As shown in figure 3A, the treatment of DC by 2-APB during 18 h decreased the percentage of quadruple positive cells (p<0.005) to 50% for all conditions. Treatments with SKF 96365 did not significantly change the marker expression level on DC whatever the maturating agents (Fig. 3B).

**Figure 4:**
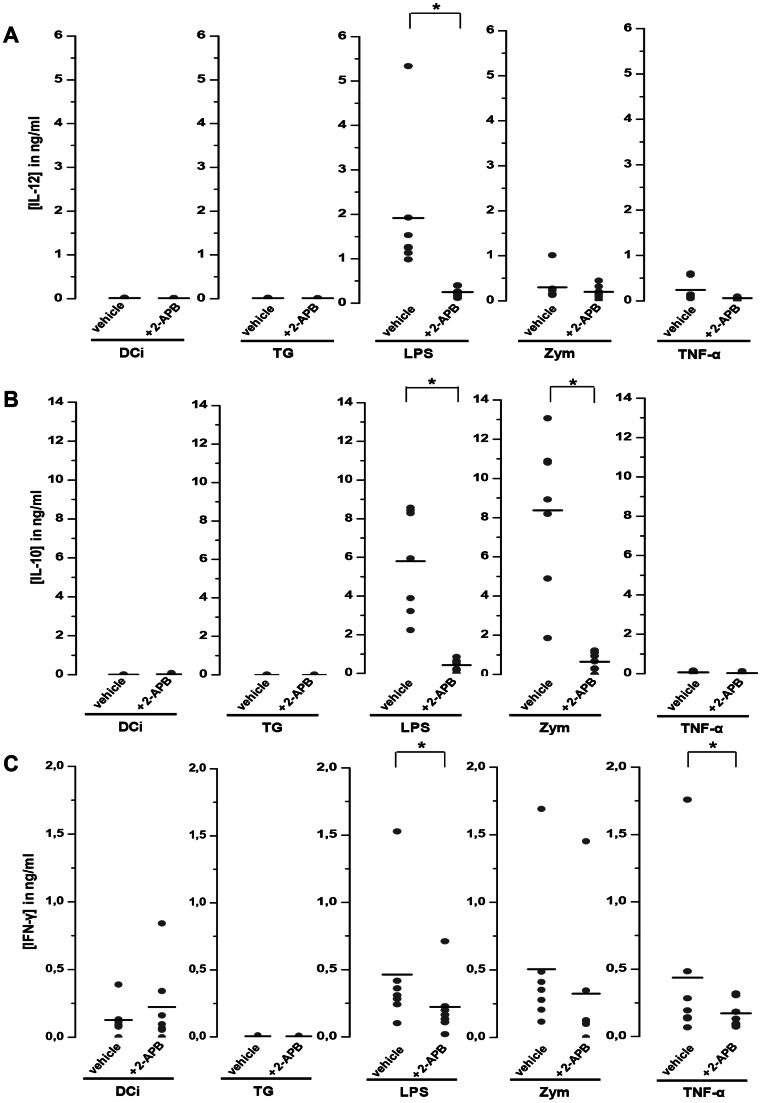
2-APB decreased the cytokine secretions in human DC. IL-12 (panel **A**), IL-10 (panel **B**) and IFN-γ (panel **C**) were measured in supernatants of DC treated by maturating agents (TG (750 nM), LPS (50 ng/ml), zymosan (25 µg/ml) or TNF-α (20 ng/ml)) for 18 h in the presence of 100 µM 2-APB. In each graph, the black line represents the mean of 7 experiments, (*p<0,05).

Cytokine secretion by DC is an essential step leading to T cell activation and polarization. We therefore evaluated DC cytokine secretion by ELISA using different inhibitors (TG or 2-APB) and maturating agents. TG-treated DC were not able to secrete any cytokine tested (Fig. 4). In our conditions, LPS-treated DC secreted IL-12, IL-10 and zymosan-treated DC secreted IL-10 (Fig. 4A and 4B). These cytokine secretions were abolished in presence of 2-APB (Fig. 4) or SKF 96365, another inhibitor of SOCE (data not shown). Secreted IFN-γ was also significantly reduced for TNF-α and LPS -DC (p<0.05) by 2-APB addition and not for zymosan-matured DC.

### 4. Both Orai1 and STIM1 proteins are expressed in DC

**Figure 5:**
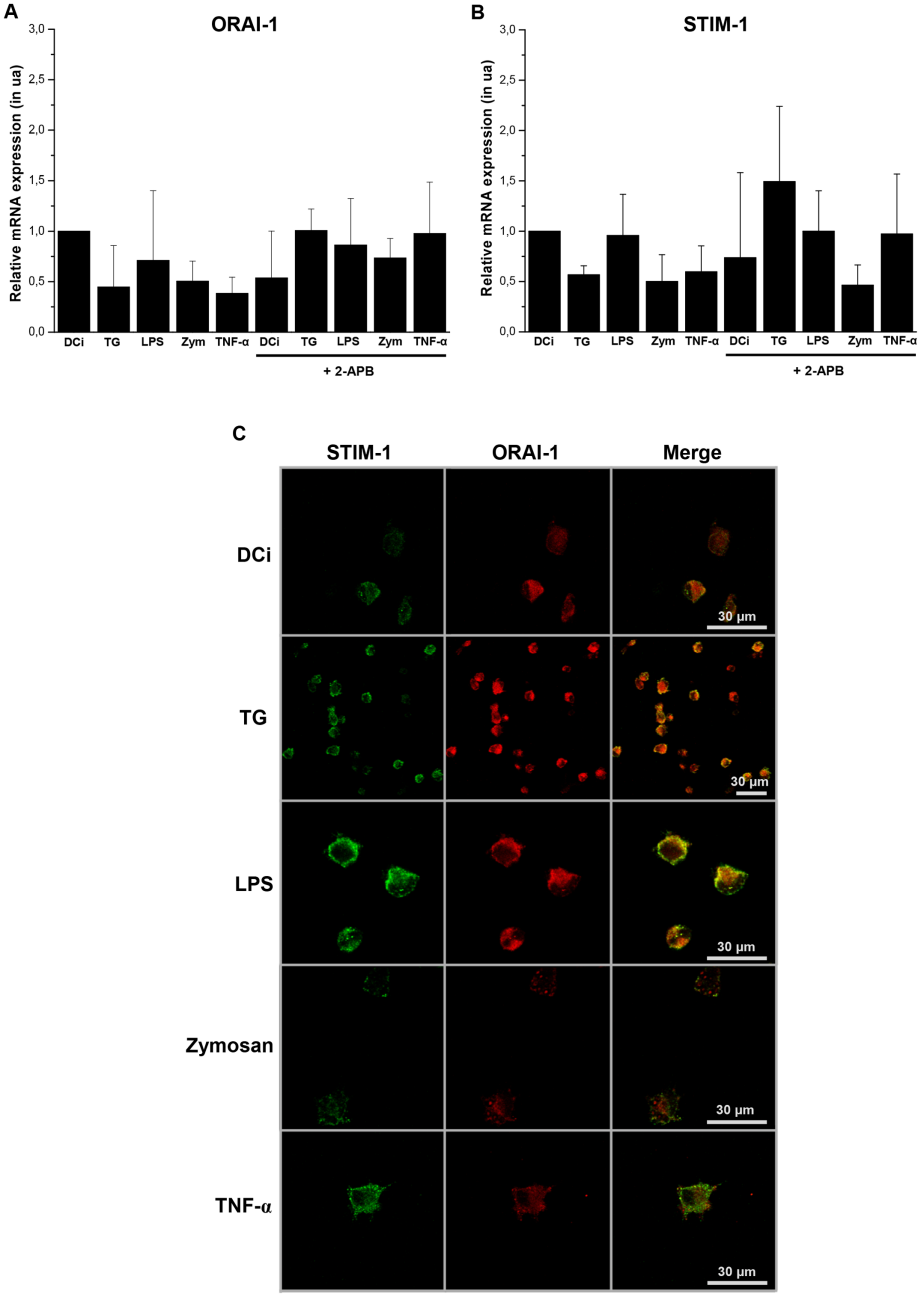
Localization of STIM-1 and Orai-1 in DC. The expression of Orai-1 and STIM-1 was analyzed at the transcriptional (**A** and **B**) or translational (**C**) level. Quantitative RT-PCR analysis of Orai-1 (**A**) and STIM-1 (**B**) mRNA showed their expression profile for different maturating stimuli (mean ± SD). The protein expressions of STIM-1 and Orai-1 were analyzed by confocal microscopy using specific antibodies coupled with a secondary antibody: Alexa Fluor®-488 for STIM-1 (**green**) and Alexa Fluor®-555 for Orai-1 (**red**). The cells were treated with maturating agents (TG (750 nM), LPS (50 ng/ml), zymosan (25 µg/ml) or TNF-α (20 ng/ml)) during 18 hours.

In order to show both Orai1 and STIM1 mRNA and proteins on DC, quantitative RT-PCR (Fig. 5A, and 5B) and confocal microscopy (Fig. 5C) analyses were conducted using specific primers and fluorescent-labeled antibodies. The Orai1 transcript was never regulated whatever the treatment conditions (Fig. 5A). The Orai1 transcript was over-expressed in all culture conditions in the presence of 2-APB (Fig. 5A). Comparatively, STIM-1 transcript expressed by DCi and its expression was unmodified by all maturating agents (Fig. 5B). Treatment with 2-APB did not affect STIM-1's expression at the transcriptional level, except in TG condition in which STIM-1 mRNA seems to be up-expressed (Fig. 5B). The Orai2 and Orai3 mRNA expressions were not modified by the different treatments as tested in real-time PCR. By the same way, STIM2 mRNAs were not observed whatever the DC maturation condition (data not shown).

We then evaluated the protein expression of Orai1 and STIM-1 in DC, at 18 h of maturation, by confocal microscopy (Fig. 5C). These two proteins appeared to be co-localized on the same cells for all conditions except for zymosan. In this condition, puncta of Orai1 (in red) were observed and STIM-1 (in green) was not detectable in the structures, as it appeared homogeneously distributed in DC (Fig. 5C).

### 5. Inhibition of both Orai1 and STIM-1 affects DC functionality

**Figure 6:**
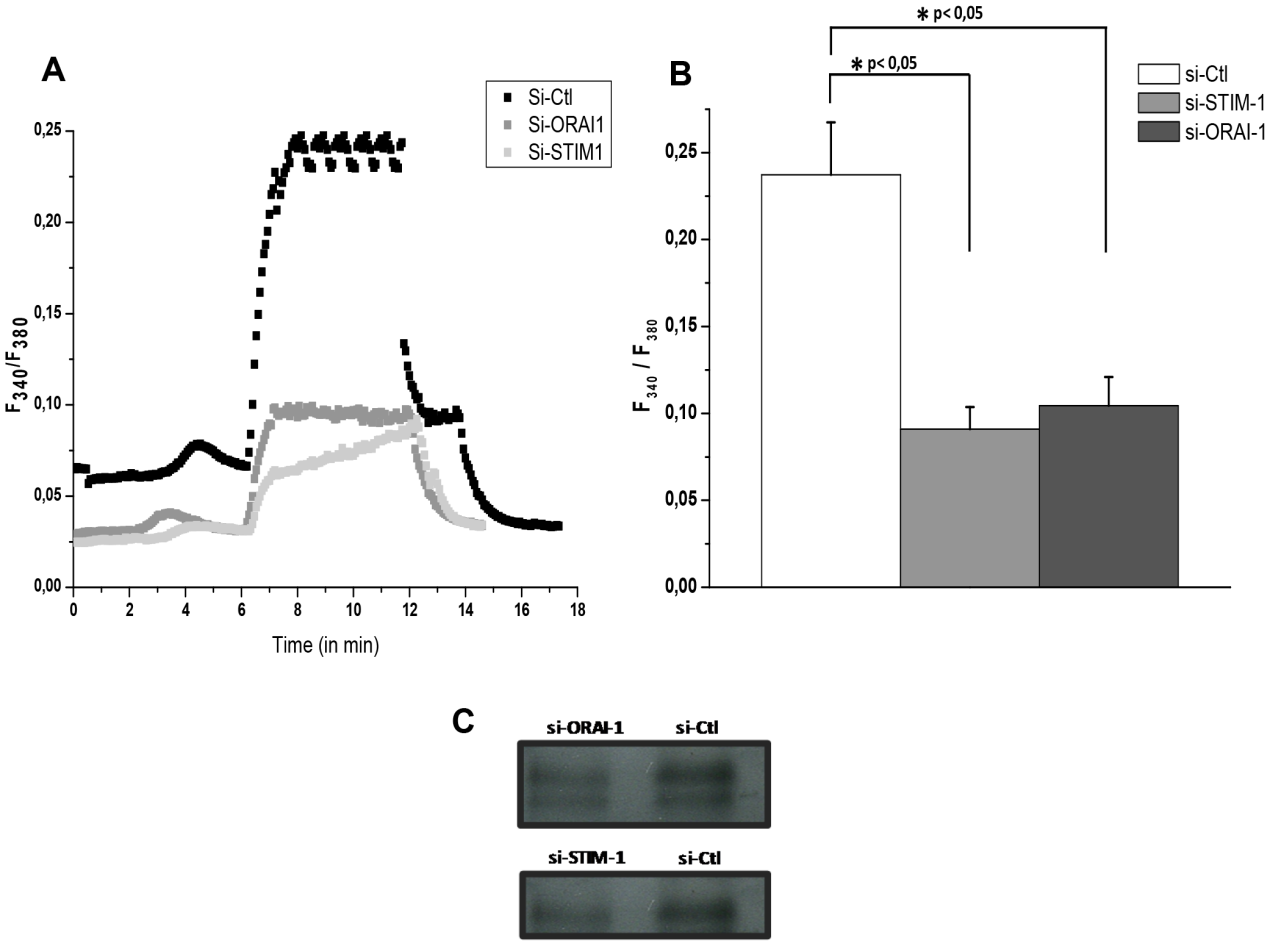
Inhibition of SOCE in DC by si-RNA (si-STIM-1 and si-Orai-1). CCE was analyzed on DC treated by specific Orai-1 (grey line) and STIM-1 (light grey line) si-RNAs for 36 h; DC were exposed to TG (in Ca^2+^-free solution) next, Ca^2+^was re-introduced (PSS, Ca^2+^ at 2 mM), a PSS solution with 2-APB (100 µM) was perfused on si-Ctl cells (black line) by microspectrofluorimetry (**A**)**.** In panel **B**, the mean amplitude of intracellular Ca^2+^ concentration is represented by bar graphs (5 experiments, mean ± SD, *p<0,05). The siRNA efficiencies were controlled by protein expression analysis using western blotting (**C**).

2APB is a non-selective blocker of store-operated channels and even it can block other channels. To determine more accurately the channels involved in the process of maturation of DCs, we chose to evaluate the involvement of Orai1 and STIM1 in DC functionality. To do so, a siRNA strategy was used against these proteins in presence of maturating agents. As we obtained an efficient inhibition (Fig. 6C), we first checked whether a SOCE was observed in the presence of a siRNA control (Black trace in Fig. 6A). Ca^2+^ (2 mM) was re-introduced in a perfused solution, after a treatment by 750 nM TG in Ca^2+^ free solution. This re-introduction provoked a sustained increase of intracellular calcium in DC (F340/F380 around 0.25). The profile of this SOCE is comparable to that obtained in figure 2A. In Si-STIM1 condition, the SOCE induced by TG treatment decreased (F340/F380 around 0.09). The SOCE amplitude was equally diminished by a treatment with Si-Orai1 (F340/F380 around 0.10) (Fig. 6B).

**Figure 7:**
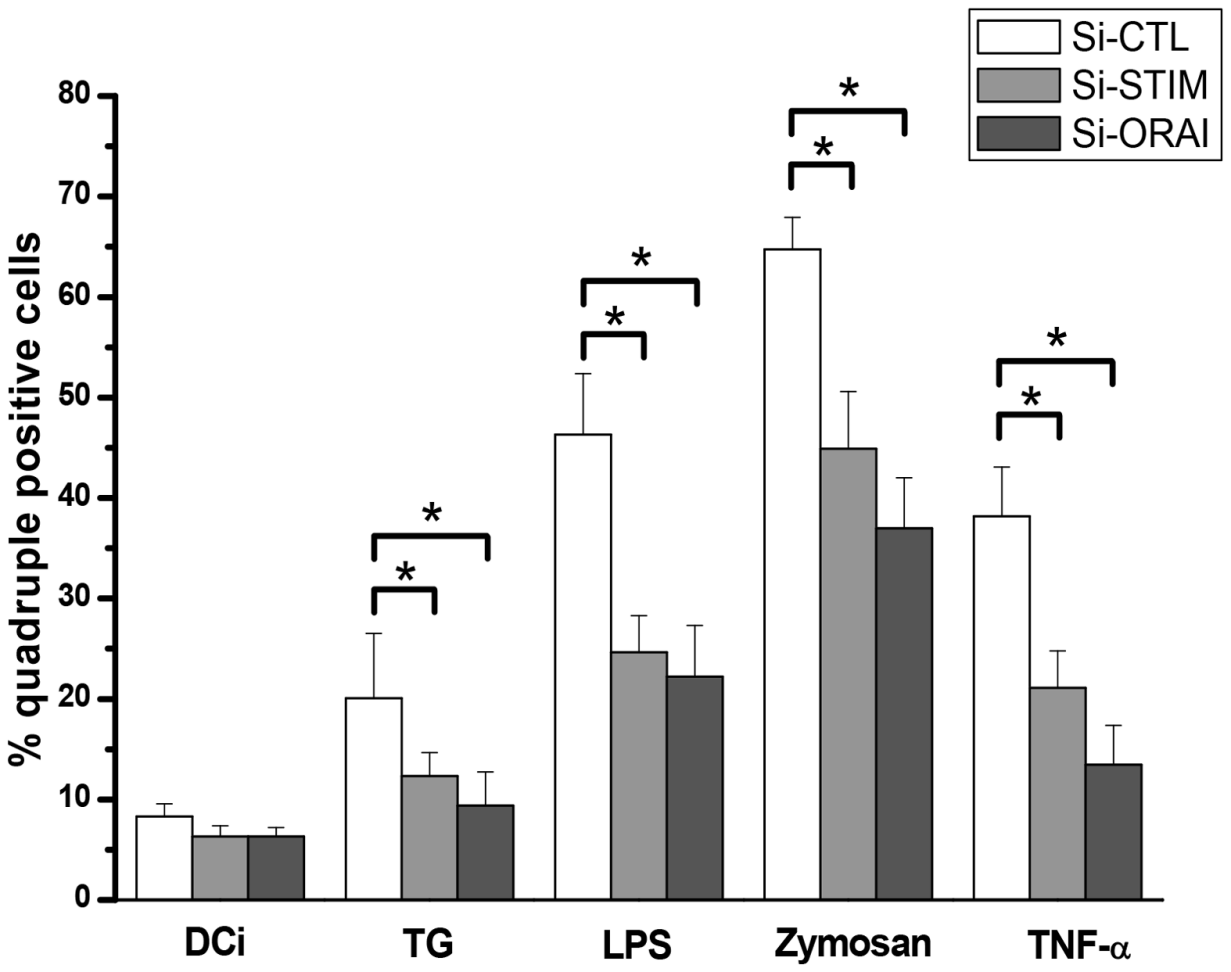
Orai-1 and STIM-1 inhibition decreased the maturation marker expressions on DC. DC treated by si-Ctl (white), by si-STIM-1 (light grey) or by si-Orai-1 (dark grey) were maturated by following maturating agents (TG at 750 nM, LPS at 50 ng/ml, zymosan at 25 µg/ml or TNF-α at 20 ng/ml) for 18 h. Then, cells were harvested and quadruple staining (CD80, CD86, CD83, CD25) was assessed by FACS analysis for each condition. The results represent the mean of percentage of quadruple positive cells from 5 independent experiments (mean ± SD, *p<0,05).

The involvement of Orai1 and STIM1 in DC maturation was also studied by the analysis of surface markers' expressions such as CD83, CD86, CD80, and HLA-DR. The increase of the expression of these markers observed in figure 3, with TG, LPS, zymosan and TNF-α was reduced by a treatment with si-STIM1 or si-Orai1 (Fig. 7).

## Discussion

The data reported here clearly underline the importance of Ca^2+^ in human dendritic cells (DC) maturation, and support an important role for Orai1 and STIM1 in DC functions. Furthermore, we report for the first time that maturating agents, such as TLR agonists or cytokines, act through a SOCE in DC. The stimulation of DC with TLR agonists induces a SOCE (Store Operated Calcium Entry) which is inhibited by 2-APB (100 µM), a known inhibitor for this kind of Ca^2+^ entry. This SOCE is involved in DC functions as demonstrated by the 2-APB inhibition of the expression of maturation markers (principally CD25 and CD83) and of the production of cytokines (IL-12, IL-10 or IFN-γ). These results were confirmed for both properties in DC through si-RNA experiments. So, we identified the STIM-1 and Orai1 channel complex as one of the main calcium pathway for DC maturation.

In mouse DC models, different SOCEs have been shown using TG and nifedipine (a L-type calcium channel inhibitor) [7]. The evidenced SOCE appears following a total Ca^2+^ depletion from the ER. Previous work has shown that CRAC channels are one of the main calcium pathways in the mouse [19]. The ion channels responsible for this depletion are IP_3_R or members of the RyR family (RyR1, RyR2 or RyR3). In murine DC, the RyR1 protein was shown to be functional contrary to what has been reported in human DC [20], [21]. In our hands, the SOCE profiles so obtained distinguished two types of maturating agents, those such as TNF-α and LPS, able to induce an irreversible SOCE with two components, and zymosan that induced a reversible SOCE with a unique component. The SOCE induced by TNF-α and LPS comprised one component that appeared 2-APB-insensitive and transient, and another, which was sustained and 2-ABP-sensitive. As for zymosan, the SOCE was reversible upon washing and less sensitive to 2-APB. This SOCE reversibility suggested that there are at least two different Ca^2+^ pathways used for SOCE in DC. Moreover, the SOCE amplitude also discriminated two categories of maturating agents, those inducing a SOCE with high amplitude such as LPS and those inducing a lower response such as TNF-α and zymosan. Dantrolene (a RyR1 inhibitor) had no effect on the SOCE induced in DC by TG, LPS, zymosan and TNF-α (data not shown). Our characterization of these SOCEs is mainly based on pharmacological studies using 2-APB, which is known to block different channels such as TRPC 1, 3, 4, 5 and 6, or SKF 96365, a potent inhibitor of TRPC 6 and 7 [22]. The SOCE induced by TG or TLR agonists in DC, was inhibited by SKF 96365 (Fig. 3B), suggesting the involvement of TRPC 6 and 7 as it has been observed in cancer cells [23]. The 2-APB specificity did not allowed the discrimination of the respective role of IP3 receptors and the membrane channels responsible of the CCE observed.

Our most striking finding was that the principal component of the SOCE induced by store-depletion appears mainly due to Orai1 and STIM1, the CRAC channel pore subunit and its activator, respectively, which are rapidly recruited to induce a Ca^2+^ influx after TLR or TNF-R activation. In mast cells, these two proteins are recruited by the activation of FcεR leading to the Ca^2+^ increase necessary to degranulation [24]. For mouse DC maturation, STIM2 seems to be the main component of the SOCE channel in the early maturation events [25]. In human DC, STIM -2 expression was not observed. But, we showed that both STIM-1 and Orai1 mRNA and protein expressions are modulated in DC treated with different maturating agents. The complex Orai1 – STIM1 appeared as mainly involved in the maturation by TNF-α and LPS but to a lower extend by zymosan. A treatment of DC with specific si-RNA (si-STIM1 or si-Orai1) induced a dramatic decrease in the SOCE's amplitude but not its abolition, indicating that only part of it might be due to the Orai1/STIM1 complex. The fact that we dramatically abolished by si-RNA STIM-1 definitely demonstrated that DC maturation and maturation marker expression are under the control a CCE involving mainly STIM-1 and Orai-1 as no changes were observed on TRPC expressions [26].

To build a functional channel, tetramers of the Orai1 protein and STIM1 Ca^2+^ sensor must interact to allow Ca^2+^ entry into the cytosol [27]. Indeed, these two proteins appeared to be co-localized in DC stimulated by the maturating agents used. When TNF-α was applied, the maturation markers were lesser expressed than in the one induced with LPS or TG, indicating that the coupling between these two proteins has demonstrated lower intensity. When zymosan is used, these proteins do not seemed not to be as clearly co-localized. The Orai1 labeling on zymosan-treated DC only showed structures as puncta. Indeed, zymosan is taken up in mouse and human DC forming phagocytosis vesicles following membrane modeling [28], [29]. Taken together the results obtained with SOCE profiles, siRNA assays and confocal microscopy, the TLR2-agonist zymosan may induce DC maturation using a different molecular calcium pathway than the TLR4-agonist LPS and TNF-α, which remains to be determined. It is therefore possible that different ion channels could be involved in affecting specific SOCE profiles as TRPC, Orai1 and STIM1 that can form heteromeric complexes [27], [30] or other members of the TRPC or ORAI families such as Orai3 in cancer cells [31].

Signaling through either TLR2 or TLR4 induces the maturation of DCi and modulates the expression of cytokines, i.e., induces IL-12, IL-10 and IFN-γ synthesis. Depending on the maturating agents used on DC, cytokine secretions appeared differently sensitive to SOCE inhibitors. Because of the DC purification method, we cannot completely spread out the possible effect of contaminating T lymphocytes. Nevertheless, the secretion of IL-12 was totally abolished by 2-APB treatment in LPS-treated DC. We could hypothesize that IL-12 secretion is related to the Ca^2+^ amplitude observed in SOCE. The IFN-γ secretion was less inhibited in zymosan-matured DC in comparison to LPS and TNF-α condition. This secretion might be more related to high and constant cytosolic Ca^2+^ level as demonstrated the continuous part of SOCE profile. We could hypothesize that according to intracellular Ca^2+^ concentration and the kinetics, various pathways might be induced leading to the cytokine secretions. Both TLR and GPCR receptor signaling have substantive roles in the regulation of DC function. Indeed, in mouse and human models, activation of TLR-4, a GPCR, provokes the recruitment of G proteins and their regulators (RGS: regulator of G protein signaling) [32]. Moreover, the cytokine production (IL-12, essentially) is regulated by GPCRs in human monocytic lineage [33]. The IFN-γ secretion is mainly dependent on calcineurin and NFAT pathways [34]. Another Ca^2+^-dependent pathway such as the Calmodulin-kinase pathway could be involved in cytokine secretion in DC [35], [36]. This pathway is involved in zymosan-activated murine macrophage for IL-10 secretion [37].

Beyond its effect on the early SOCE in DC maturation, the Orai1/STIM1 complex might have an impact on the whole immune physiology through ionic homeostasis in DC as well as in T cells [6]. The clinical phenotype associated with STIM-1 deficiency in patients is very similar to that of Orai1 deficiency, suggesting that both genes act in the same pathway and are critical for SOCE in the same tissues [17]. Additionally, deregulated ion homeostasis has been associated with pathophysiological processes in inflammation and autoimmune diseases [38], [39], [40], [41]. In fact, the Orai1/STIM1 complex because of its role in DC physiology could be a good target candidate for ion channels inhibitors in immuno-modulatory treatments.
