# Expression of Concern: The Orai-1 and STIM-1 complex controls human dendritic cell maturation

**DOI:** 10.1371/journal.pone.0231469

**Published:** 2020-04-02

**Authors:** The PLOS ONE Editors

After this article [1] was published, concerns were raised about Fig 6C:

- The upper portion of the top blot appears similar to the lower blot.
- These panels are used to demonstrate the efficiency of siRNA knockdown, but the difference between the control and experimental data in these blots is modest and the blot experiment did not include internal controls to demonstrate the relative protein abundance across lanes.

The authors provided image data in support of these results. They commented that the same number of cells is represented in each lane but they did not conduct control blots (e.g. actin) to assess relative protein levels across lanes in each blot. The image data provided (S1 File) do not match the results included in the published figure and do not demonstrate clear differences in protein expression in the experimental versus control siRNA samples.

Regarding the relative protein abundance in siRNA knockdown versus control samples, the authors acknowledged that the western blot quality was poor and they referred to supporting results in Fig 6A and 6B which indicate functional differences between experimental and control siRNA samples.

The concerns about western blot data reporting in Fig 6C are not fully resolved, and questions remain about the evidence supporting siRNA-mediated knockdown of protein expression. The article’s conclusions about roles of STIM1 and Orai1 rely on the siRNA knockdown model for which Fig 6C provides key validation data. As such, the unresolved issues have implications for whether the results reported in this article adequately support these conclusions. For these reasons, the *PLOS ONE* Editors issue this Expression of Concern.

FVR noted that the authors stand by the results in the article.

All raw data are available except those for Fig 5C and for the flow cytometry (.fcs files for Figs 1, 3 and 7) and biophysics experiments (Figs 2 and 6A).

The authors provided the following product information for reagents used in this study: siRNA ORAI-1 (h), Santa Cruz Biotechnology #sc-76001; siRNA STIM-1 Santa Cruz Biotechnology #sc-76589; anti-ORAI1 antibody, Santa Cruz Biotechnology #A47359.

## Supporting information

S1 File Image data provided in support of Fig 6C. (PPTX)
