# Supplementary material for: Expression of Concern: The Orai-1 and STIM-1 complex controls human dendritic cell maturation
Source: PLoS One. 2020 Apr 2;15(4):e0231469. doi: 10.1371/journal.pone.0231469 (PMC7117674; doi:10.1371/journal.pone.0231469)
Supplement: S1 File — (PPTX) [file pone.0231469.s001.pptx]

## Slide 1
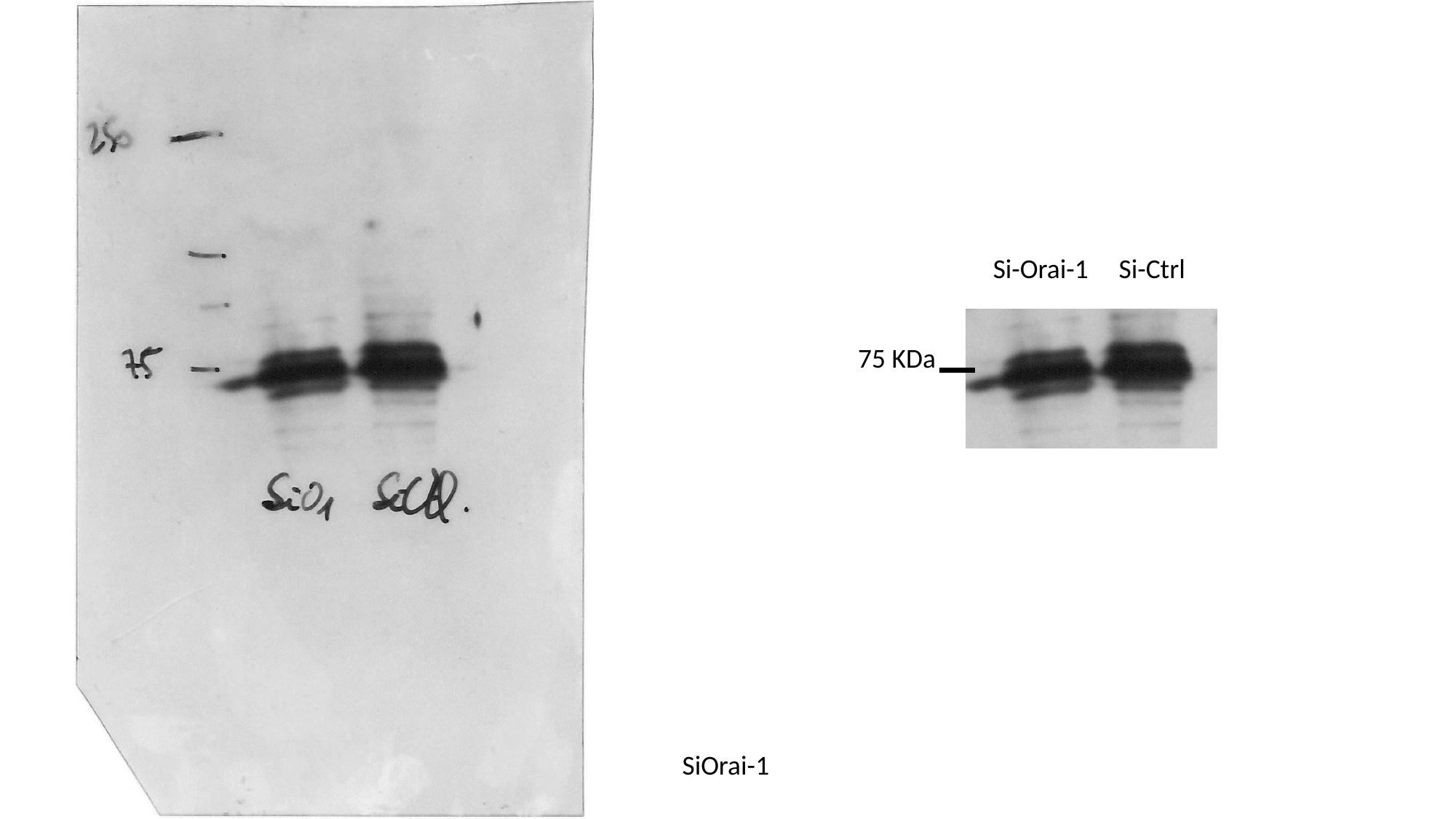

Si-Orai-1
Si-Ctrl
75 KDa
SiOrai-1

## Slide 2
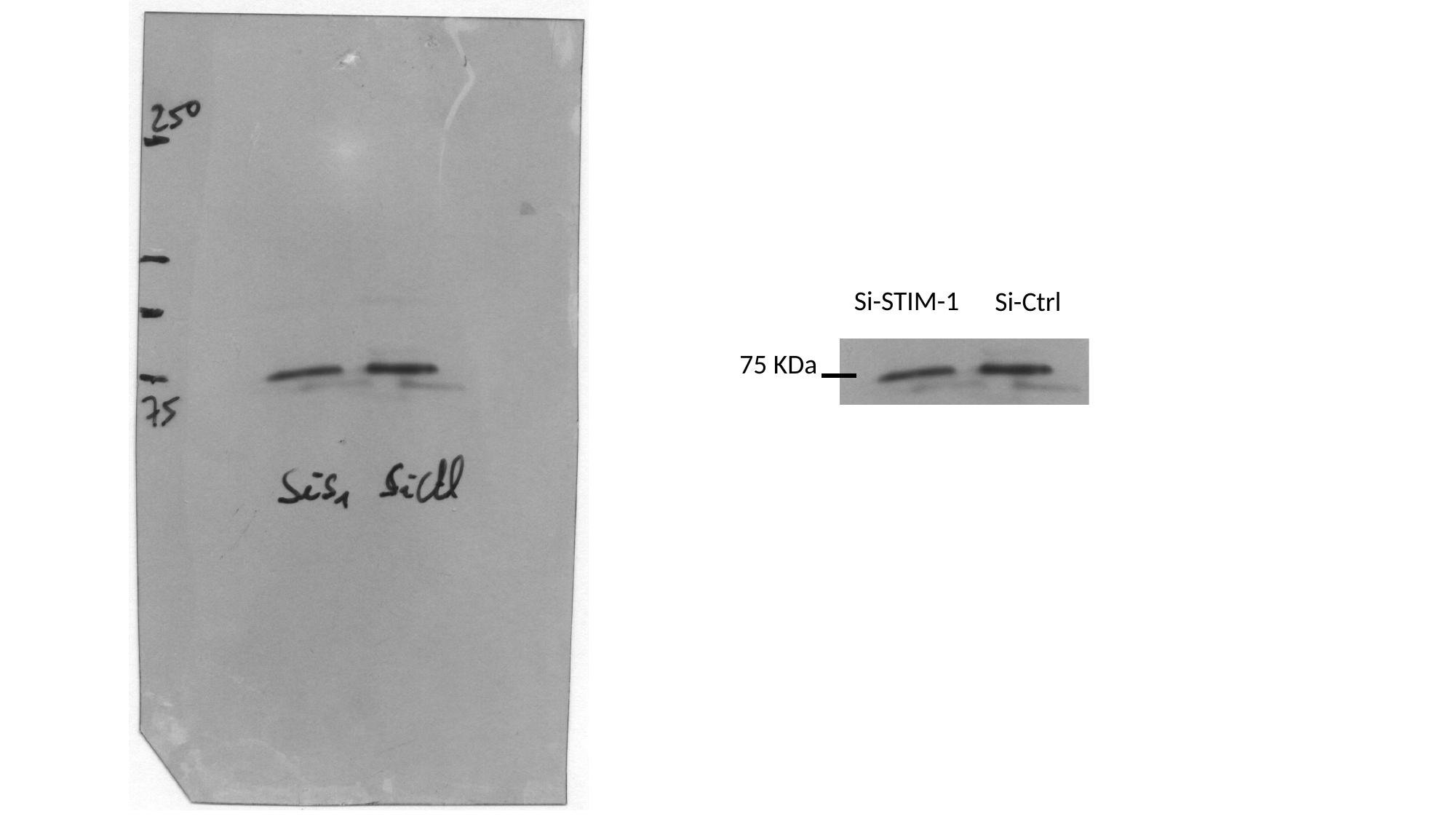

Si-STIM-1
Si-Ctrl
75 KDa

## Slide 3
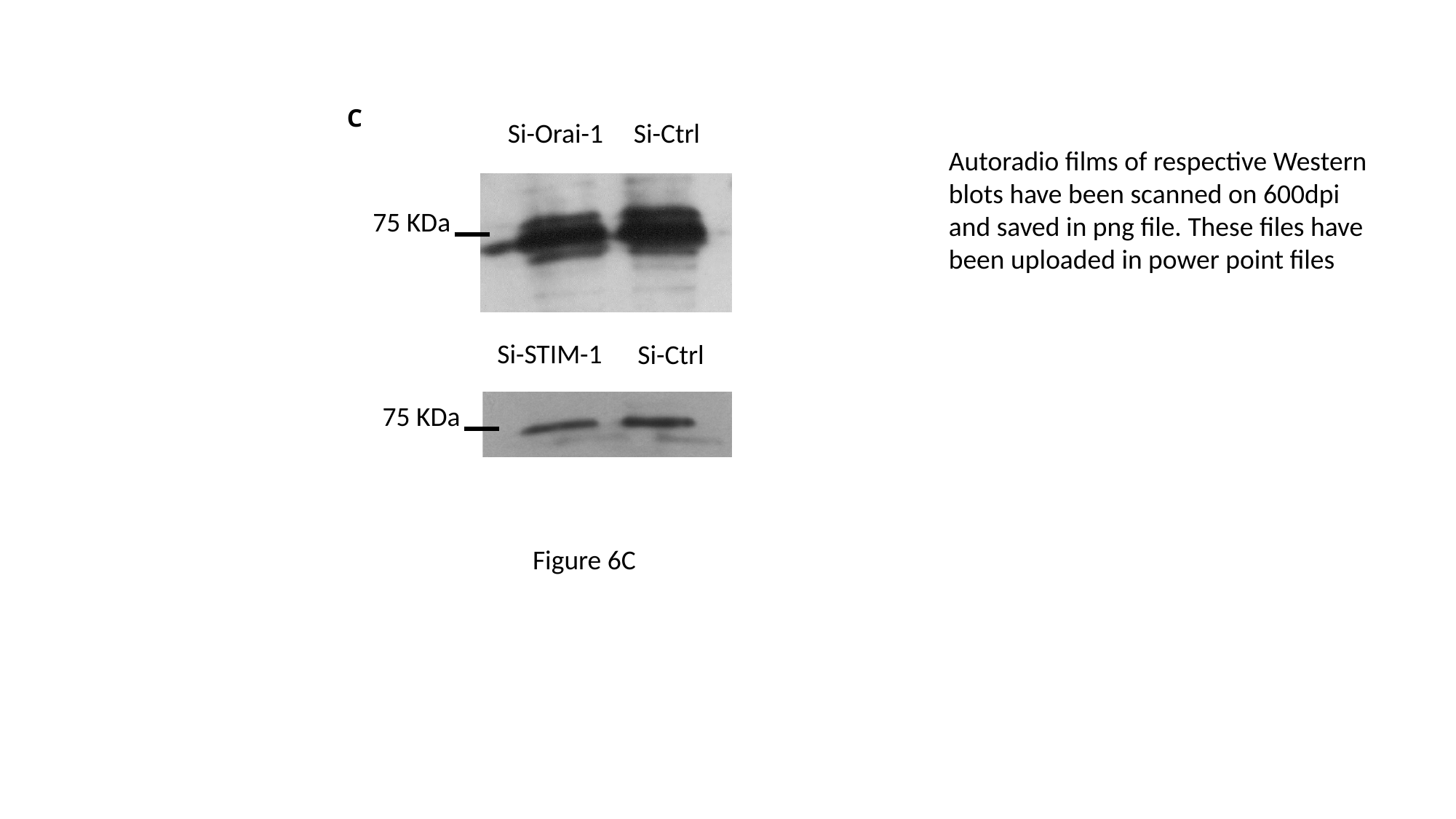

C
Si-Orai-1
Si-Ctrl
75 KDa
Si-STIM-1
Si-Ctrl
75 KDa
Autoradio films of respective Western blots have been scanned on 600dpi and saved in png file. These files have been uploaded in power point files
Figure 6C
